# Out-of-pocket expenses of patients with inflammatory bowel disease: a comparison of patient-reported outcomes across 12 European countries

**DOI:** 10.1007/s10198-022-01536-9

**Published:** 2022-10-19

**Authors:** Przemysław Holko, Paweł Kawalec, Magdalena Sajak-Szczerba, Luisa Avedano, Małgorzata Mossakowska

**Affiliations:** 1 Department of Nutrition and Drug Research, Institute of Public Health, Jagiellonian University Medical College, Skawińska 8, 31-066 Krakow, Poland; 2 European Federation of Crohn’s and Ulcerative Colitis Associations, Brussels, Belgium; 3 Polish Association Supporting People With Inflammatory Bowel Disease “J-Elita”, Warsaw, Poland; 4 International Institute of Molecular and Cell Biology, Warsaw, Poland

**Keywords:** Out-of-pocket costs, Patient-reported outcomes, Inflammatory bowel disease, I100

## Abstract

**Background:**

There is a high variability of out-of-packet patient costs of inflammatory bowel diseases (IBDs), but the issue is not widely recognised. Therefore, we compared patient costs of IBDs between 12 European countries.

**Methods:**

A questionnaire-based study was conducted among adult patients with IBD. Data on patient characteristics and out-of-pocket expenses were anonymously collected. Ordered logit regression models were used to analyse the responses provided by patients. The results were adjusted for confounders and multiplicity.

**Results:**

The questionnaires obtained from 3687 patients were analysed. Patients with comorbidities and active disease indicated higher out-of-pocket expenses than those without comorbidities and with disease in remission, respectively. Compared with other IBD, patients with ulcerative colitis indicated higher expenses on medications prescribed or recommended by physicians [odds ratio (OR) 1.99, 95% CI 1.48–2.67]. Expenses on dietary supplements, special diet or equipment, ostomy pouches, and transportation to a medical facility differed slightly between patients at different ages and were lower among men than among women (OR 0.71, 95% CI 0.54–0.93). The expenses differed significantly between countries. An adjusted mean patient cost per month varied from €77 (patient with Crohn disease in remission from Denmark) to €376 (patient with active ulcerative colitis from Romania). Compared with active disease, patients with IBD in remission had a lower out-of-pocket cost by 29–62% (€10–€22 monthly; *p* < 0.001).

**Conclusions:**

The study revealed a high relevance of the out-of-pocket cost of IBD in the context of economic evaluation and a high variability of the cost between countries.

**Supplementary Information:**

The online version contains supplementary material available at 10.1007/s10198-022-01536-9.

## Introduction

Inflammatory bowel diseases (IBDs), which primarily include Crohn disease (CD) and ulcerative colitis (UC), affect people of all ages and constitute a significant burden for the patient and the society [1, 2]. The categories of out-of-pocket cost include diet, equipment, informational material, hygiene articles, household support, patient activities, insurance deductible, transport to a medical facility, use of over-the-counter drugs, and co-payment for medications [2].

A recent systematic review [2] indicated a high variability of patient costs in Europe depending on the specific region. Only a few studies assessed patient costs, reporting a mean annual cost of $582 ($81–$1927) for CD and $497 ($181–$1341) for UC. However, the studies were limited by their focus on a single country, which makes it difficult to compare outcomes between studies. To our knowledge, to date no studies have assessed whether the different estimates of the cost between national studies are due to between-country differences in patient populations, methodology, or health-care and social care systems. European countries differ in the level of patient co-payment for medications or in the fee for specialist consultations. However, there is no evidence showing that the difference translates to significant out-of-pocket expenses or that the expenses differ by IBD type or disease activity.

To fill the existing gap in research, we aimed to compare the selected aspects of patient burden of IBD between European countries as well as to investigate these outcomes in relation to disease activity, IBD types, and other patient characteristics.

## Materials and methods

### Study design

This was a multinational online questionnaire study including adult patients diagnosed with IBD, who were invited to participate via national patient associations allied within the European Federation of Crohn’s and Ulcerative Colitis Associations (EFCCA). Participation in the study was voluntary and anonymous. No patient identifiable information was collected, and none of the questions were obligatory.

The questionnaire included questions relating to: (1) general information about the respondent (current age, age at diagnosis, sex, place of residence, IBD type: CD, UC or other IBD [e.g., microscopic colitis], comorbidities); (2) disease activity as assessed by a specialist during the last consultation (the respondent indicated disease activity as communicated to him or her by the specialist during the last consultation) and the time from the last consultation; (3) previous surgical treatment of IBD and the time from the last procedure; (4) current pharmacotherapy of IBD; (5) the patient Harvey–Bradshaw Index (P-HBI including a question on abdominal mass) in patients with CD and “other IBD” [3] or the Patient Simple Clinical Colitis Activity Index (P-SCCAI) in patients with UC [4] to obtain data on current disease activity; (6) occupational status (including disability, inability to work, education, retirement, and registered unemployment); (7) work productivity and regular activity impairment; (8) informal care; and (9) the range of monthly out-of-pocket expenses on: (a) consultations with a specialist (“consultations”); (b) medications prescribed or recommended by physicians (“medications”); (c) dietary supplements, special diet, special equipment, ostomy pouches, transportation to a medical facility (“supplements”); (d) informational materials about the disease, additional hygiene products, and others (“other expenses”). Patients could select one of the following categories of expenses: €0, €1–€49, €50–€99, €100–€149, €150–€199, €200–€249, €250 –€299, and €300 + . For the purpose of this study, euros were converted to the national currency using the current exchange rate, updated every week during the study. In this paper, we focus on out-of-pocket expenses. Informal care, work productivity and regular activity impairment were described elsewhere [5].

The study procedures were approved by the representatives of the national patient associations allied within the EFCCA as well as gastroenterologists, whose comments were collected and included in the final version of the questionnaires. The questions were prepared in English and translated by the representatives of the national patient associations. They were then translated back to check for potential errors.

The study was conducted from October 2018 to October 2019. Data from a country with at least 50 respondents with IBD were included.

### Data management

Each response was carefully evaluated, and any response not compatible with other responses was planned to be assigned as missing. Responses to the P-HBI or P-SCCAI were analysed in line with the instructions by Bennebroek Evertsz et al. [3, 4] (P-HBI score > 4 and P-SCCAI score > 5 denoting active disease). The diagnosis of IBD was based on information provided by participants.

The expenses were analysed as ordered categorical data only. However, to facilitate the interpretation of the results, an average value of the range indicated by a respondent was assigned to each expense category (e.g., the value of €74.5 was assigned to the “€50–€99” category). The sum of products of values assigned to each category and the study results (raw frequencies of each expense category or adjusted probabilities of each expense category) within each expense type (“consultations”, “medications”, “supplements” or “other expenses”) allowed to calculate the mean patient costs or differences in costs between any groups. Finally, the correlation of total out-of-pocket costs for each country with country characteristics found on EUROSTAT [8] was assessed.

All cost outcomes were presented in 2019 euros (the period to which cost data applied). The costs were assessed from the patients’ perspective.

### Statistical analysis

All study outcomes and patient characteristics were analysed descriptively and presented as a mean with standard deviation for continuous variables and as frequencies for categorical variables. Correlations were assessed using the Spearman’s *ρ* rank correlation coefficient. Comparisons between countries were performed using the Pearson *χ*^2^ test for categorical variables and the Kruskal–Wallis rank test for continuous variables.

Ordered logit regression models (with prior confirmation of proportional odds assumption) were used to analyse the responses on out-of-packet expenses. The models were fitted with a robust estimator of variance and included a categorical variable indicating IBD type, current disease activity (remission or active disease), and country, as well as covariates (sex, age, place of residence, age at diagnosis, presence of comorbidities, past surgical treatment, current biological treatment, and employment status) to control for possible confounders. Interactions between variables were included if their inclusion substantially improved the fit of the model to the data (i.e. > 10% increase in pseudo-*R*^2^ or log-likelihood). Average adjusted predictions and average marginal effects were presented as adjusted means and differences in results with standard errors (SEs) calculated using the delta method.

The analyses included all questionnaires with at least one answer. Missing data were excluded from the analysis of an outcome. The Bonferroni correction for multiple hypothesis testing (multiplicity) was incorporated. To ensure a self-explanatory attribute of the results, the *p* values and confidence intervals (CIs) were adjusted with the correction, that is, the adjusted *p* values were presented as *p* values and the CIs adjusted for multiplicity were presented as 95% CIs. The adjusted *p* value of less than 0.05 (nominal *p* value < 0.00025) was considered significant. The results were reported in adherence with the Strengthening the Reporting of Observational Studies in Epidemiology Statement [6]. Data preparation and statistical analyses were done using STATA 17SE (StataCorp, College Station, TX).

## Results

The study included 3687 patients from 12 European countries: Belgium, Bulgaria, Cyprus, Czech Republic, Denmark, Greece, Hungary, Italy, Poland, Portugal, Romania, and Spain. Patient characteristics are presented in Table 1. Missing responses occurred with a frequency of < 2% for most variables. The only exception was the comorbidity status with 8.4% missing responses. The characteristics reported in the questionnaires did not differ between complete cases and respondents with missing answers. Most patient characteristics differed between countries [see the Electronic Supplementary Material (ESM), Supplementary Table 1]. Among respondents, 1684 patients had UC (in remission, 1237; *p* = 0.081 between countries) and 1985 had CD or other IBD (in remission, 923; *p* = 0.011). Around 31% of the patients were on biological treatment (from 9.5% among patients from Poland to 57.1% among patients from Belgium; *p* < 0.001), and 61% were employed (from 48.9% among patients from Greece to 75.0% among patients from Hungary; *p* < 0.001).

**Table 1:** Characteristics of study participants

	All patients (*N* = 3687)
Age, mean (SD)	43.03 (13.76)
Male gender, *n* (%)	1241 (34.0)
Age at diagnosis, mean (SD)	29.81 (12.42)
Disease, *n* (%)	
CD	1930 (52.4)
UC	1693 (45.9)
Other IBD	63 (1.7)
Country, *n* (%)	
Belgium (BE)	128 (3.47)
Bulgaria (BG)	141 (3.82)
Cyprus (CY)	53 (1.44)
Czech Republic (CZ)	69 (1.87)
Denmark (DK)	1,253 (33.98)
Greece (GR)	264 (7.16)
Hungary (HU)	77 (2.09)
Italy (IT)	196 (5.32)
Poland (PL)	467 (12.67)
Portugal (PT)	651 (17.66)
Romania (RO)	131 (3.55)
Spain (ES)	257 (6.97)
With any comorbidities, *n* (%)	1893 (56.1)
Current pharmacotherapy, *n* (%)	
Sulfasalazine	416 (11.5)
Mesalazine	1537 (42.5)
Plain steroids	653 (18.0)
Budesonide	255 (7.0)
Azathioprine	966 (26.7)
Mercaptopurine	96 (2.7)
Methotrexate	143 (4.0)
Adalimumab	357 (9.9)
Infliximab	569 (15.7)
Golimumab	24 (0.7)
Vedolizumab	151 (4.2)
Ciclosporin	21 (0.6)
Metronidazole	147 (4.1)
Beclomethasone	19 (0.5)
Certolizumab	5 (0.1)
Ustekinumab	77 (2.1)
Past surgical treatment, *n* (%)	
Previous year	268 (7.3)
1 to 5 years ago	461 (12.6)
5 + years ago	580 (15.8)
Current disease activity, *n* (%)	
Remission	2160 (58.9)
Active disease	1509 (41.1)
UC patients with stoma, *n* (%)	132 (7.8)
Penetrating CD course, *n* (%)	595 (30.9)
Retired, *n* (%)	380 (10.3)
On a disability pension, *n* (%)	384 (10.4)
Student, *n* (%)	312 (8.5)
Registered unemployment, *n* (%)	180 (4.9)

There was no significant correlation between country and IBD type or disease activity, but a weak monotonic relationship was noted between disease activity and IBD type (i.e. a higher proportion of active CD) (*ρ* of – 0.273, *p* < 0.001).

The regression models are presented in ESM, Supplementary Table 2. Models were fitted to data from 85.1 to 86.2% of respondents (exclusion of < 15% respondents with missing responses). The raw frequencies of the expenses categories are presented in ESM, Supplementary Figs. 1–4 and Supplementary Table 3. Patients with comorbidities or active disease indicated higher out-of-pocket expenses than those without comorbidities or with disease in remission, respectively (*p* < 0.001 for both). Patients with UC indicated significantly higher categories of expenses on medications prescribed or recommended by physicians than those with other IBD [odds ratio (OR) of a higher-expense category, 1.99; 95% CI 1.48–2.67, *p* < 0.001). Expenses on dietary supplements, special diet, special equipment, ostomy pouches, and transportation to a medical facility differed slightly between patients at different ages (OR for a year increase in age 0.98; 95% CI 0.97–1.00, *p* = 0.002) and were lower among men than among women (OR 0.71; 95% CI 0.54–0.93, *p* < 0.001). The expenses differed significantly between countries (*p* < 0.001 for all). A significant interaction between disease type and country was found for the expenses on consultations with a specialist and the informational materials about the disease, additional hygiene products, and others. It appeared that UC patients from Belgium, Greece, Portugal, and Romania incurred higher expenses on consultations with a specialist than patients with other IBD, but they incurred lower expenses in the remaining countries. Unlike patients from other countries, patients with UC from Bulgaria, Cyprus, and Hungary indicated lower expenses on informational materials about the disease, additional hygiene products, and others than patients with other IBD.

**Fig. 1:**
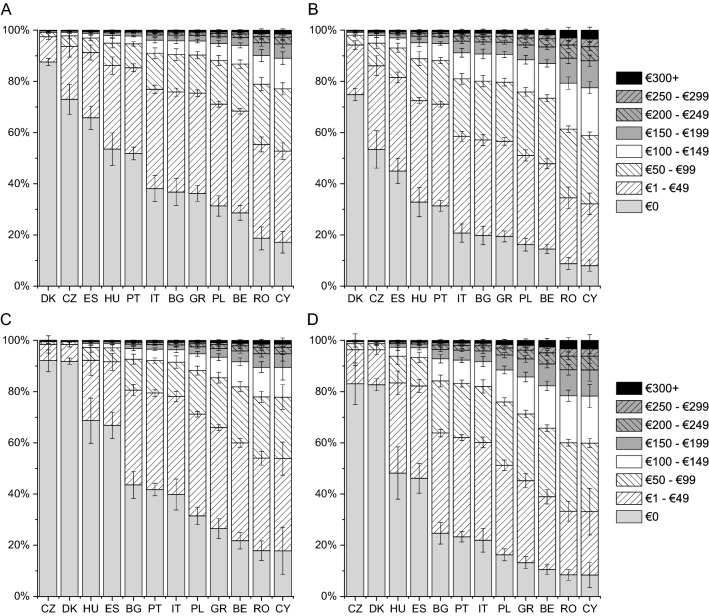
Adjusted probabilities of categories of monthly out-of-pocket expenses on consultations with a specialist among patients with Crohn disease (CD) in remission (**A**), active CD (**B**), ulcerative colitis (UC) in remission (**C**) and active UC (**D**). Error bars indicate standard errors. The difference among countries and between the remission and disease activity groups was significant with *p* < 0.001; the difference between the CD and UC groups was not significant

**Fig. 2:**
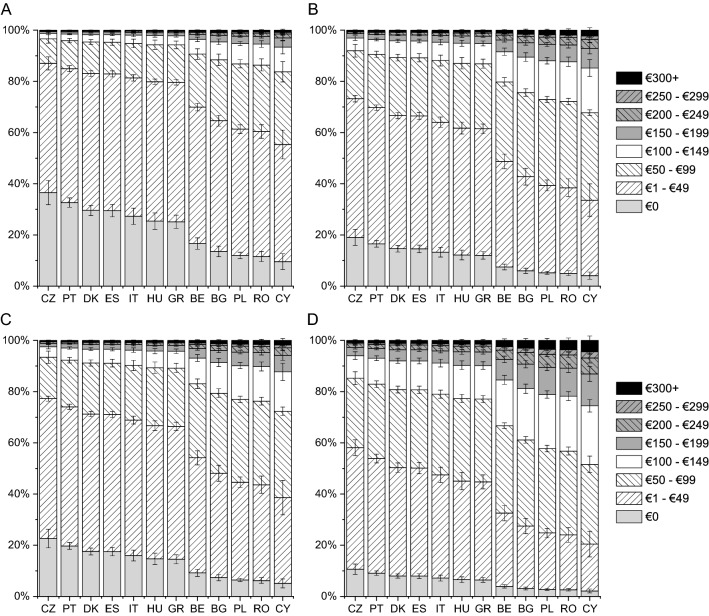
Adjusted probabilities of categories of monthly out-of-pocket expenses on medications prescribed or recommended by physicians among patients with Crohn disease (CD) in remission (**A**), active CD (**B**), ulcerative colitis (UC) in remission (**C**) and active UC (**D**). Error bars indicate standard errors. The difference among countries, between the CD and UC groups and between the remission and disease activity groups was significant with *p* < 0.001

**Fig. 3:**
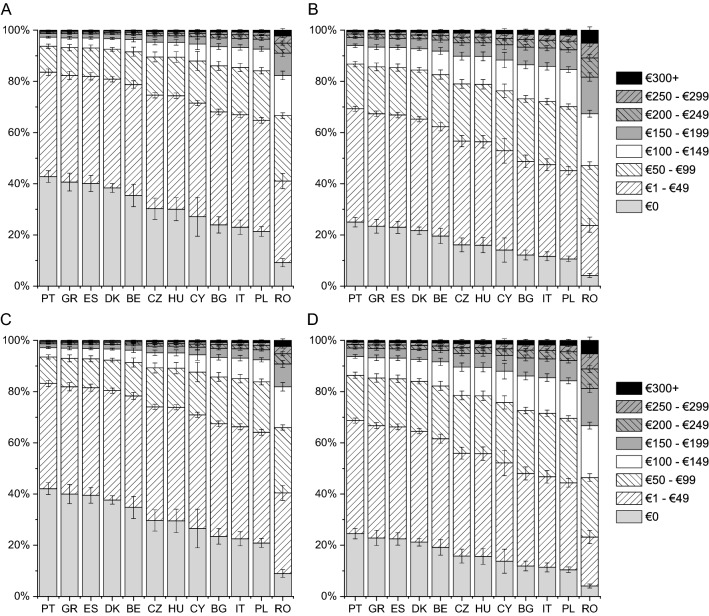
Adjusted probabilities of categories of monthly out-of-pocket expenses on dietary supplements, special diet, special equipment, ostomy pouches, transportation to the medical facility among patients with Crohn disease (CD) in remission (**A**), active CD (**B**), ulcerative colitis (UC) in remission (**C**) and active UC (**D**). Error bars indicate standard errors. The difference among countries and between the remission and disease activity groups was significant with *p* < 0.001; the difference between the CD and UC groups was not significant

**Fig. 4:**
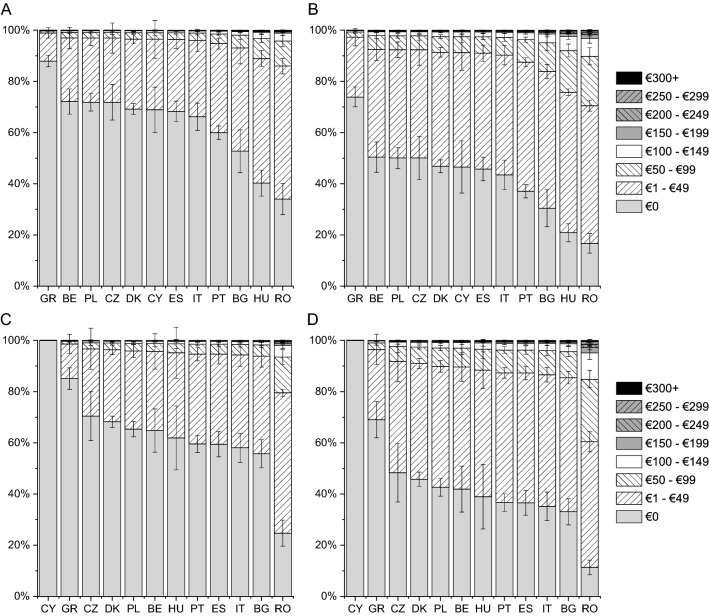
Adjusted probabilities of categories of monthly out-of-pocket expenses on informational materials about the disease, additional hygiene products and others among patients with Crohn disease (CD) in remission (**A**), active CD (**B**), ulcerative colitis (UC) in remission (**C**) and active UC (**D**). Error bars indicate standard errors. The difference among countries and between the remission and disease activity groups was significant with *p* < 0.001; the difference between the CD and UC groups was not significant

The lowest expenses on consultations with a specialist were incurred by patients from Denmark and Czech Republic and the highest by those from Romania and Cyprus (Fig. 1). The difference in monthly expenses on consultations with a specialist was as high as €60 among patients with disease in remission (Denmark vs Cyprus) and as high as €90 among patients with active disease (Czech Republic vs Cyprus). According to respondents, the expenses on medications prescribed or recommended by physicians were the lowest in Czech Republic and Portugal and the highest in Romania and Cyprus, with the monthly difference between those countries ranging from €30 to €60 (Fig. 2). The expenses on supplements, special diet, special equipment, ostomy pouches, and transportation to a medical facility were similar between countries except for Romania, where patients with active disease incurred an expense of €120 a month (Fig. 3). Patients from Romania indicated the highest expenses on informational materials about the disease, additional hygiene products, and others (CD: by €25–€35 higher than those from Greece; UC: by €35–€55 than those from Cyprus; Fig. 4). Compared with patients with active disease, patients with IBD in remission had a lower out-of-pocket cost by €16 (SE of 0.96, “consultations”), €21 (SE of 1.28, “medications”), €22 (SE of 1.21, “supplements”), and €10 (SE of 0.62, “other expenses”) (all, *p* < 0.001). This corresponds to reduction in patient cost of 29–62% depending on the country and expenses type.

The mean patient cost obtained with an average valuation of each category is presented in Table 2. The adjusted mean patient cost (all categories combined) ranged from €76.64 (CD in remission, Denmark) to €375.68 (active UC, Romania).

**Table 2:** The out-of-pocket patient expenses per patient per month by country, disease type (Crohn disease, CD or ulcerative colitis, UC) and disease activity (adjusted mean with SE in brackets; in 2019 euros)

Country	IBD	Consultations		Medications		Supplements		Other expenses	
		Remission	Active disease	Remission	Active disease	Remission	Active disease	Remission	Active disease
BE	CD	45.46 (2.70)	72.38 (4.16)	43.51 (2.70)	66.02 (4.16)	34.14 (2.70)	54.76 (4.16)	9.13 (2.70)	17.81 (4.16)
	UC	55.93 (3.96)	86.23 (5.95)	59.93 (3.96)	87.96 (5.95)	34.75 (3.96)	55.61 (5.95)	11.85 (3.96)	22.00 (5.95)
BG	CD	36.23 (3.24)	59.70 (4.69)	48.87 (3.24)	73.33 (4.69)	47.51 (3.24)	73.11 (4.69)	16.77 (3.24)	29.22 (4.69)
	UC	30.17 (2.75)	51.09 (4.13)	66.76 (2.75)	96.74 (4.13)	48.27 (2.75)	74.14 (4.13)	15.47 (2.75)	27.35 (4.13)
CY	CD	65.53 (6.20)	98.49 (8.74)	58.61 (6.20)	86.24 (8.74)	43.19 (6.20)	67.27 (8.74)	10.29 (6.20)	19.64 (8.74)
	UC	64.05 (12.24)	96.62 (16.37)	78.91 (12.24)	111.93 (16.37)	43.90 (12.24)	68.25 (16.37)	0.00 (12.24)	0.00 (16.37)
CZ	CD	11.80 (2.44)	22.92 (3.24)	25.08 (2.44)	40.17 (3.24)	39.40 (2.44)	62.08 (3.24)	9.22 (2.44)	17.96 (3.24)
	UC	3.19 (1.77)	7.06 (3.37)	36.05 (1.77)	55.63 (3.37)	40.07 (1.77)	63.01 (3.37)	9.73 (1.77)	18.77 (3.37)
DK	CD	5.09 (0.61)	10.88 (1.03)	29.82 (0.61)	46.85 (1.03)	31.53 (0.61)	51.08 (1.03)	10.20 (0.61)	19.50 (1.03)
	UC	3.27 (0.51)	7.23 (0.98)	42.19 (0.51)	64.19 (0.98)	32.10 (0.51)	51.89 (0.98)	10.54 (0.51)	20.02 (0.98)
ES	CD	15.51 (1.92)	29.00 (2.56)	29.94 (1.92)	47.02 (2.56)	30.05 (1.92)	48.97 (2.56)	10.54 (1.92)	20.01 (2.56)
	UC	14.96 (2.15)	28.11 (2.90)	42.34 (2.15)	64.41 (2.90)	30.60 (2.15)	49.76 (2.90)	13.93 (2.15)	25.10 (2.90)
GR	CD	36.74 (2.18)	60.41 (3.36)	33.56 (2.18)	52.13 (3.36)	29.63 (2.18)	48.37 (3.36)	3.79 (2.18)	8.49 (3.36)
	UC	48.39 (3.61)	76.31 (5.43)	47.06 (3.61)	70.86 (5.43)	30.17 (3.61)	49.15 (5.43)	4.71 (3.61)	10.25 (5.43)
HU	CD	22.85 (2.84)	40.34 (3.90)	33.34 (2.84)	51.83 (3.90)	39.61 (2.84)	62.38 (3.90)	22.91 (2.84)	38.04 (3.90)
	UC	14.00 (3.62)	26.56 (4.72)	46.77 (3.62)	70.48 (4.72)	40.29 (3.62)	63.30 (4.72)	12.95 (3.62)	23.65 (4.72)
IT	CD	34.91 (3.04)	57.84 (4.40)	31.67 (3.04)	49.46 (4.40)	48.92 (3.04)	75.00 (4.40)	11.30 (3.04)	21.18 (4.40)
	UC	33.38 (3.38)	55.68 (5.09)	44.59 (3.38)	67.50 (5.09)	49.71 (3.38)	76.05 (5.09)	14.50 (3.38)	25.93 (5.09)
PL	CD	42.08 (3.01)	67.79 (4.45)	52.26 (3.01)	77.86 (4.45)	51.62 (3.01)	78.59 (4.45)	9.22 (3.01)	17.96 (4.45)
	UC	41.94 (2.71)	67.60 (4.22)	71.02 (2.71)	102.12 (4.22)	52.43 (2.71)	79.66 (4.22)	11.61 (2.71)	21.65 (4.22)
PT	CD	23.99 (1.34)	42.04 (2.03)	27.61 (1.34)	43.74 (2.03)	27.95 (1.34)	45.98 (2.03)	13.71 (1.34)	24.78 (2.03)
	UC	31.70 (1.62)	53.29 (2.71)	39.32 (1.62)	60.22 (2.71)	28.48 (1.62)	46.73 (2.71)	13.88 (1.62)	25.03 (2.71)
RO	CD	61.98 (5.92)	94.01 (8.27)	53.22 (5.92)	79.14 (8.27)	84.98 (5.92)	120.35 (8.27)	26.71 (5.92)	43.51 (8.27)
	UC	63.81 (5.45)	96.33 (8.01)	72.22 (5.45)	103.62 (8.01)	86.10 (5.45)	121.68 (8.01)	34.02 (5.45)	54.05 (8.01)

**Fig. 5:**
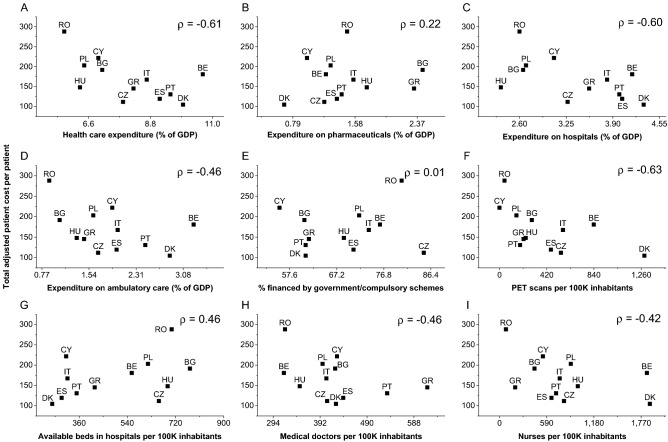
Total adjusted patient cost (in 2019 euros) per patient per month as a function of: **A** health-care expenditure (share of gross domestic product, GDP); **B** expenditure on pharmaceuticals and other medical non-durable goods (share of GDP); **C** expenditure on hospitals (share of GDP); **D** expenditure on ambulatory care (share of GDP); **E** share of health-care expenditure financed by government/compulsory schemes; **F** number of positron emission tomography (PET) scans per 100,000 inhabitants; **G** available beds in hospitals per 100,000 inhabitants; **H** medical doctors per 100,000 inhabitants; **I** Nurses and midwives per 100,000 inhabitants. Spearman’s *ρ* rank correlation coefficient is presented in each graph

Total out-of-pocket costs for each country correlated with some characteristics of the health-care system (from the strongest to the weakest corelation, Fig. 5): number of positron emission tomography scans per 100,000 inhabitants; health-care expenditure as a share of gross domestic product (GDP); expenditure on hospitals (share of GDP); expenditure on ambulatory care (share of GDP); available beds in hospitals per 100,000 inhabitants; medical doctors per 100,000 inhabitants; nurses and midwives per 100,000 inhabitants; expenditure on pharmaceuticals and other medical non-durable goods (share of GDP); and the share of health-care expenditure financed by government/compulsory schemes.

## Discussion

The results of the study suggest that the induction and maintenance of remission in all participants with active IBD might reduce patient cost by 29–62% (i.e. €10 to €22 monthly, depending on the expense category), thus ensuring an additional argument to highly prioritise effective IBD treatments. The significant variability between countries in the amount of impact of IBD activity was observed, with a marginal cost per month (all categories combined) ranging from €52 among CD patients from Denmark to €120 among UC patients from Romania (€69.43, SE 2.10 across all countries). Nevertheless, since data were collected once for each participant, the relationship between the outcomes and disease activity may require additional confirmation in a longitudinal study.

To our best knowledge, this is the first study reporting the patient cost of IBD exclusively among patients from Belgium, Bulgaria, Cyprus, Czech Republic, Denmark, Greece, Hungary, Italy, Portugal, and Romania [2]. The strengths of the study include a large sample size as well as a multinational background and diversity of the respondents, obtained by direct enrolment without additional restrictions (e.g. during specialist consultation or hospitalisation). The limitations include possible sampling bias (the sample may not be representative of the general IBD population) and the fact that self-reported information can be influenced by recall, response, or social desirability biases. The sample size differed between countries, which might bias the adjusted estimates towards results among patients from countries with the largest sample sizes. Finally, we asked respondents about IBD-attributable costs only. However, the results of our study show that patients with comorbidities indicate higher expenses than those without comorbidities. This may be caused either by the negative effect of comorbidities on the course of IBD or by the fact that patients do not distinguish between expenses on IBD and those on comorbidity. A systematic review by Attauabi et al. [7] found that the simultaneous presence of other immune-mediated inflammatory diseases affects the course of IBD. Furthermore, the current average exchange rates used during the study to convert EUR to other currencies could affect the results, since the conversion was not adjusted for purchasing power.

We did not measure specific aspects that may be responsible for the difference in patient expenses among the included countries. Lifestyle and socioeconomic factors that were not evenly distributed among countries may be responsible. However, those aspects were not measured in our study. Differences in health care or social policies can also be responsible. In fact, a moderate negative association is easily observed between the amount of patient expenses and some country characteristics (total health-care expenditure, expenditure on hospitals, and the number of positron emission tomography scans per 100,000 inhabitants). Interestingly, the amount of patient expenses was not correlated with the share of health-care expenditure financed by government/compulsory schemes (Fig. 5).

van Linschoten et al. [2] identified eight studies [9–16] reporting patient cost and updated the results of each study to 2018 US dollars (as presented below). Two studies were conducted among patients from countries included in our study: a study among 200 patients with CD from Poland (2015) [10] and a study among 635 patients with CD from Spain (1997) [11]. The mean patient cost in the Polish and Spanish studies was around $70 and 7$ per patient per month, respectively. In the present study, the overall monthly patient cost ranged from €59 (remission) to €86 (active disease) among CD patients from Poland, and from €87 (remission) to €155 (active disease) among CD patients from Spain (Table 2). Importantly, the Spanish study included travel cost only [11], while the Polish study included similar expense categories as those in our study [10]. Moreover, the proportion of patients with active disease in the Polish study [10] was comparable to that reported in the current study (52.5% and 53.5%, respectively, among patients with CD), but our studies differ in the proportion of patients using biological treatment (14% [10] and 31%, respectively, among patients from all countries). This discrepancy probably results from limited access to biological treatment in Poland (9.5% patients from Poland used biological treatment in our study).

Other studies identified by van Linschoten et al. [2] indicated a slightly higher patient cost among patients with CD than among patients with UC: $23 vs $15 per month in England (overall out-of-pocket expenses) [9], $53 vs $38 per month in Serbia (cost of transport, nutrition, alternative therapy, others) [12], $161 vs $112 per month in Germany (cost of special diet, household support, transport, others) [14], $35 vs $26 per month in a cross-sectional study from the Netherlands (deductible, travel, over-the-counter drugs) [15], and $20 vs $16 per month in a longitudinal study from the Netherlands (deductible, travel, over-the-counter drugs) [16]. In our study, the cost of the “consultations”, “supplements”, and “other expenses” categories did not differ between IBD types. Compared with other IBD, a higher cost of medications prescribed or recommended by physicians among patients with UC was observed with a marginal cost of €16.04 (SE 1.15) per month across all countries (from €11 among patients with disease in remission from the Czech Republic to €26 among patients with active disease from Cyprus, Table 2). The majority of the studies either did not include patient co-payment for medications [12, 14–16] or included it in the composite category of patient cost (i.e. patients were asked about overall IBD-attributable cost, which may have affected the precision of the responses) [9]. Additionally, in all those studies, active disease was more prevalent among CD patients than among UC patients. Moreover, in line with the objectives, none of the studies had adjusted their results for the differences in characteristics between CD and UC patients. Our study confirmed that patients with active disease incurred higher expenses than those with disease in remission. Therefore, the higher patient cost in previous studies may be due to a higher percentage of CD patients with more severe disease as compared with UC patients. Thus, the higher adjusted expenses on medication among UC patients in our study may indicate that the level of funding of the drugs differs between IBD types in the included European countries.

The results of our study indicate the importance of patient cost in the context of economic analyses and can be used to inform the cost-effectiveness models of new treatments or to support the decision-making process relating to health policies, resource allocation, and patient care. Future multinational studies should consider between-country variability when assessing the patient burden of IBD because these aspects can be largely determined by country-dependent health-care and societal policies.

## Supplementary Information

Below is the link to the electronic supplementary material.

Supplementary file1 (DOCX 356 kb)

## Data Availability

All data are presented in the manuscript or the supplementary materials. The dataset is available from the corresponding author upon reasonable request.
