# Supplementary material for: Out-of-pocket expenses of patients with inflammatory bowel disease: a comparison of patient-reported outcomes across 12 European countries
Source: Eur J Health Econ. 2022 Oct 19;24(7):1073–83. doi: 10.1007/s10198-022-01536-9 (PMC10406674; doi:10.1007/s10198-022-01536-9)
Supplement: Supplementary file 1 — Supplementary file1 (DOCX 356 kb) [file 10198_2022_1536_MOESM1_ESM.docx]

**Supplementary materials**

Supplementary Table 1. Characteristics of all study participants by country.

|  | All | Belgium | Bulgaria | Cyprus | Czech R. | Denmark | Greece | Spain | Poland | Portugal | Romania | Hungary | Italy | P value |
| --- | --- | --- | --- | --- | --- | --- | --- | --- | --- | --- | --- | --- | --- | --- |
| N | 3,687 | 128 | 141 | 53 | 69 | 1,253 | 264 | 257 | 467 | 651 | 131 | 77 | 196 | - |
| Age, mean (SD) | 43.03 (13.76) | 42.38 (13.75) | 41.18 (10.90) | 37.15 (12.74) | 37.90 (11.87) | 48.46 (14.61) | 39.78 (11.96) | 46.12 (11.62) | 37.90 (11.71) | 39.44 (12.04) | 38.20 (12.67) | 35.91 (10.24) | 43.78 (13.93) | <0.0001 |
| Male gender, n (%) | 1241 (34.0) | 43 (33.9) | 52 (37.7) | 24 (47.1) | 24 (34.8) | 369 (29.7) | 100 (38.5) | 99 (39.1) | 195 (42.0) | 183 (28.5) | 43 (32.8) | 26 (33.8) | 83 (42.4) | 0.0003 |
| Age at diagnosis, mean (SD) | 29.81 (12.42) | 27.68 (11.33) | 32.84 (11.33) | 26.88 (11.95) | 25.49 (10.55) | 32.26 (14.02) | 28.77 (11.93) | 29.15 (11.39) | 29.10 (11.14) | 27.51 (10.63) | 30.45 (12.95) | 25.97 (10.29) | 28.45 (11.29) | <0.0001 |
| *Place of residence, n (%)* |  |  |  |  |  |  |  |  |  |  |  |  |  |  |
| City with a population of ≥100 thousands | 1545 (42.2) | 39 (30.5) | 80 (58.0) | 27 (51.9) | 19 (27.9) | 405 (32.6) | 172 (65.2) | 91 (35.6) | 234 (50.1) | 304 (47.0) | 66 (51.2) | 48 (62.3) | 60 (30.9) | <0.0001 |
| City with a population of <100 thousands | 1242 (33.9) | 35 (27.3) | 50 (36.2) | 11 (21.2) | 25 (36.8) | 375 (30.2) | 63 (23.9) | 164 (64.1) | 156 (33.4) | 205 (31.7) | 45 (34.9) | 18 (23.4) | 95 (49.0) |  |
| Village | 876 (23.9) | 54 (42.2) | 8 (5.8) | 14 (26.9) | 24 (35.3) | 463 (37.3) | 29 (11.0) | 1 (0.4) | 77 (16.5) | 138 (21.3) | 18 (14.0) | 11 (14.3) | 39 (20.1) |  |
| *Disease, n (%)* |  |  |  |  |  |  |  |  |  |  |  |  |  |  |
| CD | 1930 (52.4) | 86 (67.2) | 54 (38.3) | 33 (62.3) | 41 (59.4) | 603 (48.1) | 179 (67.8) | 142 (55.3) | 186 (39.8) | 395 (60.7) | 61 (46.9) | 57 (74.0) | 93 (47.5) | <0.0001 |
| UC | 1693 (45.9) | 40 (31.3) | 84 (59.6) | 20 (37.7) | 27 (39.1) | 629 (50.2) | 84 (31.8) | 109 (42.4) | 273 (58.5) | 242 (37.2) | 66 (50.8) | 20 (26.0) | 99 (50.5) |  |
| Other IBD | 63 (1.7) | 2 (1.6) | 3 (2.1) | 0 (0.0) | 1 (1.5) | 21 (1.7) | 1 (0.4) | 6 (2.3) | 8 (1.7) | 14 (2.2) | 3 (2.3) | 0 (0.0) | 4 (2.0) |  |
| *Comorbidities, n (%)* |  |  |  |  |  |  |  |  |  |  |  |  |  |  |
| Any | 1893 (56.1) | 65 (55.1) | 80 (60.6) | 28 (58.3) | 43 (67.2) | 574 (51.0) | 144 (60.3) | 141 (59.0) | 287 (64.9) | 341 (57.2) | 57 (46.7) | 32 (46.4) | 101 (55.5) | 0.0071 |
| Diseases of the joints, musculoskeletal system or connective tissue | 527 (15.6) | 17 (14.4) | 11 (8.3) | 5 (10.4) | 15 (23.4) | 123 (10.9) | 50 (20.9) | 37 (15.5) | 84 (19.0) | 133 (22.3) | 5 (4.1) | 6 (8.7) | 41 (22.5) | <0.0001 |
| Diseases of the eye | 256 (7.6) | 7 (5.9) | 7 (5.3) | 4 (8.3) | 8 (12.5) | 43 (3.8) | 26 (10.9) | 19 (8.0) | 54 (12.2) | 60 (10.1) | 13 (10.7) | 2 (2.9) | 13 (7.1) | 0.0001 |
| Liver or bile duct diseases | 217 (6.4) | 2 (1.7) | 17 (12.9) | 1 (2.1) | 6 (9.4) | 54 (4.8) | 14 (5.9) | 14 (5.9) | 49 (11.1) | 33 (5.5) | 13 (10.7) | 4 (5.8) | 10 (5.5) | 0.0035 |
| Other than IBD diseases of the digestive system | 242 (7.2) | 13 (11.0) | 14 (10.6) | 4 (8.3) | 5 (7.8) | 56 (5.0) | 26 (10.9) | 23 (9.6) | 37 (8.4) | 35 (5.9) | 6 (4.9) | 7 (10.1) | 16 (8.8) | >0.9999 |
| Diseases of the skin and subcutaneous tissue | 472 (14.0) | 13 (11.0) | 16 (12.1) | 1 (2.1) | 15 (23.4) | 167 (14.8) | 27 (11.3) | 38 (15.9) | 67 (15.2) | 87 (14.6) | 19 (15.6) | 2 (2.9) | 20 (11.0) | >0.9999 |
| Diseases of the bones - osteoporosis | 356 (10.6) | 13 (11.0) | 18 (13.6) | 8 (16.7) | 12 (18.8) | 80 (7.1) | 42 (17.6) | 36 (15.1) | 42 (9.5) | 55 (9.2) | 22 (18.0) | 4 (5.8) | 24 (13.2) | 0.0001 |
| Kidney diseases | 145 (4.3) | 5 (4.2) | 10 (7.6) | 2 (4.2) | 4 (6.3) | 33 (2.9) | 5 (2.1) | 15 (6.3) | 20 (4.5) | 32 (5.4) | 9 (7.4) | 0 (0.0) | 10 (5.5) | >0.9999 |
| Tumor (cancer) | 63 (1.9) | 3 (2.5) | 2 (1.5) | 0 (0.0) | 1 (1.6) | 20 (1.8) | 3 (1.3) | 3 (1.3) | 11 (2.5) | 13 (2.2) | 2 (1.6) | 1 (1.5) | 4 (2.2) | >0.9999 |
| Diseases of the blood or blood-forming organs or certain disorders involving the immune system | 148 (4.4) | 8 (6.8) | 13 (9.9) | 1 (2.1) | 5 (7.8) | 26 (2.3) | 9 (3.8) | 10 (4.2) | 38 (8.6) | 23 (3.9) | 6 (4.9) | 1 (1.5) | 8 (4.4) | 0.0007 |
| Endocrine, nutritional or metabolic diseases | 324 (9.6) | 9 (7.6) | 17 (12.9) | 6 (12.5) | 7 (10.9) | 66 (5.9) | 38 (15.9) | 23 (9.6) | 72 (16.3) | 46 (7.7) | 16 (13.1) | 10 (14.5) | 14 (7.7) | <0.0001 |
| Mental and behavioral disorders | 237 (7.0) | 4 (3.4) | 7 (5.3) | 7 (14.6) | 8 (12.5) | 87 (7.7) | 40 (16.7) | 25 (10.5) | 19 (4.3) | 26 (4.4) | 3 (2.5) | 6 (8.7) | 5 (2.8) | <0.0001 |
| Diseases of the nervous system | 168 (5.0) | 3 (2.5) | 10 (7.6) | 6 (12.5) | 5 (7.8) | 34 (3.0) | 12 (5.0) | 9 (3.8) | 26 (5.9) | 47 (7.9) | 5 (4.1) | 5 (7.3) | 6 (3.3) | 0.0873 |
| Diseases of the ear | 76 (2.3) | 1 (0.9) | 3 (2.3) | 0 (0.0) | 3 (4.7) | 25 (2.2) | 7 (2.9) | 6 (2.5) | 7 (1.6) | 17 (2.9) | 0 (0.0) | 1 (1.5) | 6 (3.3) | >0.9999 |
| Diseases of the circulatory system | 184 (5.5) | 1 (0.9) | 6 (4.6) | 2 (4.2) | 2 (3.1) | 54 (4.8) | 7 (2.9) | 15 (6.3) | 48 (10.9) | 24 (4.0) | 6 (4.9) | 5 (7.3) | 14 (7.7) | 0.0078 |
| Diseases of the respiratory system | 297 (8.8) | 10 (8.5) | 9 (6.8) | 2 (4.2) | 7 (10.9) | 112 (10.0) | 15 (6.3) | 22 (9.2) | 35 (7.9) | 58 (9.7) | 14 (11.5) | 1 (1.5) | 12 (6.6) | >0.9999 |
| Diseases of the genitourinary system | 118 (3.5) | 2 (1.7) | 7 (5.3) | 2 (4.2) | 7 (10.9) | 12 (1.1) | 8 (3.4) | 9 (3.8) | 29 (6.6) | 19 (3.2) | 7 (5.7) | 3 (4.4) | 13 (7.1) | 0.0001 |
| Congenital malformations, deformations and/or chromosomal abnormalities | 18 (0.5) | 2 (1.7) | 1 (0.8) | 0 (0.0) | 1 (1.6) | 6 (0.5) | 0 (0.0) | 0 (0.0) | 3 (0.7) | 2 (0.3) | 2 (1.6) | 1 (1.5) | 0 (0.0) | >0.9999 |
| Infectious or parasitic diseases | 64 (1.9) | 4 (3.4) | 4 (3.0) | 4 (8.3) | 1 (1.6) | 10 (0.9) | 3 (1.3) | 5 (2.1) | 22 (5.0) | 6 (1.0) | 3 (2.5) | 1 (1.5) | 1 (0.6) | 0.0005 |
| *Current pharmacotherapy, n (%)* |  |  |  |  |  |  |  |  |  |  |  |  |  |  |
| Any | 3074 (84.9) | 112 (88.9) | 112 (79.4) | 43 (82.7) | 54 (79.4) | 938 (77.0) | 236 (91.8) | 225 (87.6) | 418 (90.5) | 580 (90.3) | 127 (98.5) | 55 (74.3) | 174 (89.7) | <0.0001 |
| Sulfasalazine | 416 (11.5) | 12 (9.5) | 46 (32.6) | 5 (9.6) | 6 (8.8) | 94 (7.7) | 11 (4.3) | 10 (3.9) | 98 (21.2) | 84 (13.1) | 30 (23.3) | 11 (14.9) | 9 (4.6) | <0.0001 |
| Mesalazine | 1537 (42.5) | 45 (35.7) | 56 (39.7) | 22 (42.3) | 26 (38.2) | 410 (33.7) | 80 (31.1) | 91 (35.4) | 297 (64.3) | 279 (43.5) | 80 (62.0) | 30 (40.5) | 121 (62.4) | <0.0001 |
| Plain steroids | 653 (18.0) | 16 (12.7) | 34 (24.1) | 6 (11.5) | 19 (27.9) | 382 (31.4) | 13 (5.1) | 14 (5.5) | 28 (6.1) | 51 (7.9) | 63 (48.8) | 7 (9.5) | 20 (10.3) | <0.0001 |
| Budesonide | 255 (7.0) | 11 (8.7) | 10 (7.1) | 1 (1.9) | 9 (13.2) | 81 (6.7) | 12 (4.7) | 6 (2.3) | 36 (7.8) | 40 (6.2) | 27 (20.9) | 15 (20.3) | 7 (3.6) | <0.0001 |
| Azathioprine | 966 (26.7) | 30 (23.8) | 33 (23.4) | 15 (28.9) | 14 (20.6) | 298 (24.5) | 82 (31.9) | 71 (27.6) | 144 (31.2) | 203 (31.6) | 34 (26.4) | 20 (27.0) | 22 (11.3) | 0.0008 |
| Mercaptopurine | 96 (2.7) | 7 (5.6) | 0 (0.0) | 1 (1.9) | 0 (0.0) | 46 (3.8) | 1 (0.4) | 7 (2.7) | 27 (5.8) | 3 (0.5) | 3 (2.3) | 0 (0.0) | 1 (0.5) | <0.0001 |
| Methotrexate | 143 (4.0) | 4 (3.2) | 1 (0.7) | 1 (1.9) | 1 (1.5) | 75 (6.2) | 17 (6.6) | 12 (4.7) | 8 (1.7) | 16 (2.5) | 5 (3.9) | 0 (0.0) | 3 (1.6) | 0.0031 |
| Adalimumab | 357 (9.9) | 22 (17.5) | 18 (12.8) | 0 (0.0) | 6 (8.8) | 53 (4.4) | 26 (10.1) | 57 (22.2) | 7 (1.5) | 93 (14.5) | 43 (33.3) | 9 (12.2) | 23 (11.9) | <0.0001 |
| Infliximab | 569 (15.7) | 21 (16.7) | 2 (1.4) | 9 (17.3) | 9 (13.2) | 221 (18.1) | 49 (19.1) | 37 (14.4) | 27 (5.8) | 132 (20.6) | 37 (28.7) | 4 (5.4) | 21 (10.8) | <0.0001 |
| Golimumab | 24 (0.7) | 1 (0.8) | 1 (0.7) | 0 (0.0) | 0 (0.0) | 7 (0.6) | 5 (2.0) | 5 (2.0) | 0 (0.0) | 3 (0.5) | 1 (0.8) | 0 (0.0) | 1 (0.5) | >0.9999 |
| Vedolizumab | 151 (4.2) | 13 (10.3) | 2 (1.4) | 1 (1.9) | 3 (4.4) | 30 (2.5) | 13 (5.1) | 20 (7.8) | 8 (1.7) | 39 (6.1) | 1 (0.8) | 5 (6.8) | 16 (8.3) | <0.0001 |
| Ciclosporin | 21 (0.6) | 0 (0.0) | 3 (2.1) | 0 (0.0) | 0 (0.0) | 5 (0.4) | 0 (0.0) | 2 (0.8) | 1 (0.2) | 4 (0.6) | 3 (2.3) | 0 (0.0) | 3 (1.6) | >0.9999 |
| Metronidazole | 147 (4.1) | 2 (1.6) | 8 (5.7) | 2 (3.9) | 1 (1.5) | 55 (4.5) | 3 (1.2) | 4 (1.6) | 21 (4.6) | 15 (2.3) | 30 (23.3) | 1 (1.4) | 5 (2.6) | <0.0001 |
| Beclomethasone | 19 (0.5) | 2 (1.6) | 0 (0.0) | 0 (0.0) | 0 (0.0) | 4 (0.3) | 0 (0.0) | 1 (0.4) | 1 (0.2) | 1 (0.2) | 0 (0.0) | 0 (0.0) | 10 (5.2) | <0.0001 |
| Certolizumab | 5 (0.1) | 0 (0.0) | 0 (0.0) | 0 (0.0) | 0 (0.0) | 2 (0.2) | 1 (0.4) | 0 (0.0) | 1 (0.2) | 0 (0.0) | 1 (0.8) | 0 (0.0) | 0 (0.0) | >0.9999 |
| Ustekinumab | 77 (2.1) | 15 (11.9) | 0 (0.0) | 1 (1.9) | 4 (5.9) | 14 (1.2) | 8 (3.1) | 17 (6.6) | 1 (0.2) | 5 (0.8) | 0 (0.0) | 1 (1.4) | 11 (5.7) | <0.0001 |
| Any biological treatment | 1123 (31.0) | 72 (57.1) | 23 (16.3) | 11 (21.2) | 21 (30.9) | 296 (24.3) | 102 (39.7) | 132 (51.4) | 44 (9.5) | 266 (41.4) | 66 (51.2) | 18 (24.3) | 72 (37.1) | <0.0001 |
| *Past surgical treatment, n (%)* |  |  |  |  |  |  |  |  |  |  |  |  |  |  |
| previous year | 268 (7.3) | 13 (10.2) | 6 (4.3) | 1 (2.0) | 8 (11.6) | 87 (7.0) | 22 (8.5) | 13 (5.1) | 35 (7.5) | 43 (6.6) | 13 (10.0) | 8 (10.5) | 19 (9.7) | <0.0001 |
| 1 to 5 years ago | 461 (12.6) | 20 (15.6) | 10 (7.1) | 1 (2.0) | 14 (20.3) | 156 (12.5) | 33 (12.7) | 43 (17.0) | 63 (13.6) | 64 (9.9) | 22 (16.9) | 12 (15.8) | 23 (11.8) |  |
| 5+ years ago | 580 (15.8) | 22 (17.2) | 15 (10.6) | 2 (4.0) | 10 (14.5) | 246 (19.7) | 23 (8.9) | 53 (21.0) | 33 (7.1) | 120 (18.5) | 4 (3.1) | 16 (21.1) | 36 (18.5) |  |
| *Disease activity at last clinical assessment, n (%)* |  |  |  |  |  |  |  |  |  |  |  |  |  |  |
| Remission | 1989 (54.3) | 64 (50.0) | 55 (39.3) | 28 (54.9) | 39 (56.5) | 772 (62.0) | 170 (64.9) | 162 (63.3) | 250 (54.2) | 256 (39.4) | 60 (47.6) | 48 (63.2) | 85 (43.4) | <0.0001 |
| Active disease | 1490 (40.7) | 60 (46.9) | 73 (52.1) | 21 (41.2) | 29 (42.0) | 399 (32.0) | 86 (32.8) | 83 (32.4) | 188 (40.8) | 360 (55.4) | 59 (46.8) | 27 (35.5) | 105 (53.6) |  |
| 'don't know'/ ‘don’t remember’ | 182 (5.0) | 4 (3.1) | 12 (8.6) | 2 (3.9) | 1 (1.5) | 75 (6.0) | 6 (2.3) | 11 (4.3) | 23 (5.0) | 34 (5.2) | 7 (5.6) | 1 (1.3) | 6 (3.1) |  |
| *Time of the last assessment of disease activity, n (%)* |  |  |  |  |  |  |  |  |  |  |  |  |  |  |
| previous month | 1307 (35.9) | 53 (41.4) | 39 (28.3) | 14 (28.6) | 32 (46.4) | 316 (25.5) | 122 (46.9) | 85 (33.7) | 201 (43.5) | 289 (44.5) | 50 (40.0) | 27 (35.5) | 79 (40.7) | <0.0001 |
| 1-2 months ago | 637 (17.5) | 24 (18.8) | 17 (12.3) | 11 (22.5) | 12 (17.4) | 224 (18.1) | 34 (13.1) | 53 (21.0) | 84 (18.2) | 104 (16.0) | 18 (14.4) | 13 (17.1) | 43 (22.2) |  |
| 2-3 months ago | 581 (16.0) | 22 (17.2) | 22 (15.9) | 11 (22.5) | 12 (17.4) | 182 (14.7) | 41 (15.8) | 75 (29.8) | 63 (13.6) | 99 (15.2) | 22 (17.6) | 7 (9.2) | 25 (12.9) |  |
| 3+ months ago | 1118 (30.7) | 29 (22.7) | 60 (43.5) | 13 (26.5) | 13 (18.8) | 518 (41.8) | 63 (24.2) | 39 (15.5) | 114 (24.7) | 158 (24.3) | 35 (28.0) | 29 (38.2) | 47 (24.2) |  |
| *Current disease activity, n (%)* |  |  |  |  |  |  |  |  |  |  |  |  |  |  |
| Remission | 2160 (58.9) | 52 (40.6) | 91 (64.5) | 20 (39.2) | 23 (33.8) | 825 (66.1) | 138 (52.9) | 143 (55.6) | 282 (60.5) | 362 (55.8) | 72 (56.3) | 43 (56.6) | 109 (55.6) | <0.0001 |
| Active disease | 1509 (41.1) | 76 (59.4) | 50 (35.5) | 31 (60.8) | 45 (66.2) | 423 (33.9) | 123 (47.1) | 114 (44.4) | 184 (39.5) | 287 (44.2) | 56 (43.8) | 33 (43.4) | 87 (44.4) |  |
| *Current activity of CD or other IBD, n (%)* |  |  |  |  |  |  |  |  |  |  |  |  |  |  |
| Remission | 923 (46.5) | 28 (31.8) | 29 (50.9) | 11 (34.4) | 9 (21.4) | 331 (53.2) | 85 (47.5) | 61 (41.2) | 84 (43.3) | 188 (46.2) | 32 (50.8) | 29 (51.8) | 36 (37.1) | 0.0109 |
| Active disease | 1062 (53.5) | 60 (68.2) | 28 (49.1) | 21 (65.6) | 33 (78.6) | 291 (46.8) | 94 (52.5) | 87 (58.8) | 110 (56.7) | 219 (53.8) | 31 (49.2) | 27 (48.2) | 61 (62.9) |  |
| P-HBI score, mean (SD) | 6.00 (5.45) | 7.56 (5.25) | 5.28 (4.17) | 6.38 (4.37) | 7.79 (5.11) | 5.56 (5.47) | 5.53 (4.28) | 7.54 (8.25) | 6.22 (5.14) | 5.63 (4.88) | 6.02 (6.20) | 5.43 (5.59) | 6.85 (4.76) | 0.0022 |
| *Current activity of UC, n (%)* |  |  |  |  |  |  |  |  |  |  |  |  |  |  |
| Remission | 1237 (73.5) | 24 (60.0) | 62 (73.8) | 9 (47.4) | 14 (53.9) | 494 (78.9) | 53 (64.6) | 82 (75.2) | 198 (72.8) | 174 (71.9) | 40 (61.5) | 14 (70.0) | 73 (73.7) | 0.0806 |
| Active disease | 447 (26.5) | 16 (40.0) | 22 (26.2) | 10 (52.6) | 12 (46.2) | 132 (21.1) | 29 (35.4) | 27 (24.8) | 74 (27.2) | 68 (28.1) | 25 (38.5) | 6 (30.0) | 26 (26.3) |  |
| P-SCCAI score (UC), mean (SD) | 3.78 (3.24) | 4.80 (4.01) | 3.62 (3.55) | 5.68 (3.71) | 5.42 (4.29) | 3.33 (2.93) | 4.23 (3.20) | 3.74 (2.98) | 4.05 (3.44) | 3.79 (3.06) | 3.95 (3.40) | 4.30 (4.54) | 4.26 (3.45) | 0.8370 |
| UC patients with stoma, n (%) | 132 (7.8) | 1 (2.5) | 0 (0.0) | 2 (10.0) | 1 (3.7) | 54 (8.6) | 10 (12.1) | 17 (15.6) | 11 (4.0) | 13 (5.4) | 10 (15.2) | 4 (20.0) | 9 (9.1) | 0.0163 |
| Penetrating CD course, n (%) | 595 (30.9) | 25 (29.4) | 15 (28.3) | 5 (17.9) | 20 (48.8) | 207 (33.7) | 43 (25.9) | 25 (18.0) | 55 (29.0) | 129 (32.2) | 15 (25.0) | 20 (37.0) | 36 (39.1) | 0.4391 |
| Employed, n (%) | 2455 (67.0) | 73 (57.0) | 104 (73.8) | 32 (64.0) | 48 (69.6) | 833 (66.9) | 127 (48.9) | 171 (66.5) | 349 (74.9) | 472 (73.0) | 74 (57.8) | 57 (75.0) | 115 (59.0) | <0.0001 |
| During short-term absence from work, n (%) | 407 (11.0) | 13 (10.2) | 28 (19.9) | 5 (9.4) | 9 (13.0) | 116 (9.3) | 15 (5.7) | 27 (10.5) | 49 (10.5) | 97 (14.9) | 13 (9.9) | 10 (13.0) | 25 (12.8) | 0.0576 |
| Retired, n (%) | 380 (10.3) | 15 (11.7) | 7 (5.0) | 2 (3.8) | 2 (2.9) | 203 (16.2) | 14 (5.3) | 23 (9.0) | 28 (6.0) | 38 (5.8) | 25 (19.1) | 2 (2.6) | 21 (10.7) | <0.0001 |
| On a disability pension, n (%) | 384 (10.4) | 6 (4.7) | 29 (20.6) | 2 (3.8) | 25 (36.2) | 175 (14.0) | 42 (15.9) | 24 (9.3) | 33 (7.1) | 14 (2.2) | 9 (6.9) | 14 (18.2) | 11 (5.6) | <0.0001 |
| Unable to work due to IBD, n (%) | 343 (9.3) | 36 (28.1) | 16 (11.4) | 4 (7.6) | 8 (11.6) | 88 (7.0) | 23 (8.7) | 26 (10.1) | 56 (12.0) | 32 (4.9) | 16 (12.2) | 10 (13.0) | 28 (14.3) | <0.0001 |
| With disability certificate | 523 (14.2) | 13 (10.2) | 9 (6.4) | 2 (3.8) | 7 (10.1) | 18 (1.4) | 58 (22.0) | 90 (35.0) | 161 (34.5) | 82 (12.6) | 8 (6.1) | 4 (5.2) | 71 (36.2) | <0.0001 |
| Student, n (%) | 312 (8.5) | 5 (3.9) | 9 (6.4) | 5 (9.4) | 8 (11.6) | 87 (6.9) | 27 (10.2) | 19 (7.4) | 40 (8.6) | 65 (10.0) | 18 (13.7) | 7 (9.1) | 22 (11.2) | >0.9999 |
| Registered unemployment, n (%) | 180 (4.9) | 2 (1.6) | 5 (3.6) | 3 (5.7) | 3 (4.4) | 32 (2.6) | 37 (14.0) | 19 (7.4) | 11 (2.4) | 47 (7.2) | 0 (0.0) | 2 (2.6) | 19 (9.7) | <0.0001 |
| Not registered unemployment, n (%) | 160 (4.3) | 1 (0.8) | 13 (9.2) | 2 (3.8) | 1 (1.5) | 32 (2.6) | 19 (7.2) | 6 (2.3) | 26 (5.6) | 35 (5.4) | 9 (6.9) | 3 (3.9) | 13 (6.6) | 0.0122 |
| Number of consultations with clinician in previous month, mean (SD) | 1.84 (3.47) | 2.25 (2.42) | 2.25 (4.35) | 1.69 (2.19) | 1.83 (2.29) | 1.23 (3.11) | 1.98 (2.72) | 5.43 (5.46) | 1.54 (2.88) | 1.56 (3.07) | 2.45 (4.35) | 1.79 (2.52) | 1.63 (3.04) | <0.0001 |
| Length of hospital stay in previous month, mean (SD) | 0.89 (2.85) | 1.12 (2.41) | 1.38 (3.75) | 1.29 (4.08) | 0.52 (1.98) | 0.72 (2.11) | 0.61 (2.43) | 0.67 (3.58) | 0.78 (2.97) | 1.23 (3.42) | 1.90 (3.96) | 0.30 (1.07) | 0.78 (2.81) | <0.0001 |

Supplementary Table 2. Ordered logistic regression models for out-of-pocket expenses.

|  | ‘Consultations’, OR (95% CI) | ‘Medications’, OR (95% CI) | ‘Supplements’, OR (95% CI) | ‘Other expenses’, OR (95% CI) |
| --- | --- | --- | --- | --- |
| a year increase in age | 0.9917 (0.9750 - 1.0086) | 1.0013 (0.9870 - 1.0158) | 0.9844 (0.9709 - 0.9981) | 0.9933 (0.9779 - 1.0090) |
| sex: men vs women | 0.9835 (0.7292 - 1.3265) | 0.8938 (0.6883 - 1.1606) | 0.7109 (0.5409 - 0.9343) | 0.8209 (0.6061 - 1.1118) |
| place: city <100K vs city ≥100K | 0.9778 (0.7037 - 1.3586) | 0.9703 (0.7227 - 1.3027) | 1.0086 (0.7554 - 1.3467) | 0.9876 (0.7165 - 1.3613) |
| place: village vs city ≥100K | 1.1386 (0.7867 - 1.6479) | 1.0637 (0.7544 - 1.4999) | 0.9843 (0.7090 - 1.3665) | 0.9107 (0.6298 - 1.3167) |
| a year increase in age at diagnosis | 1.0086 (0.9913 - 1.0262) | 1.0006 (0.9858 - 1.0155) | 0.9973 (0.9833 - 1.0114) | 1.0079 (0.9913 - 1.0247) |
| Comorbidity: any vs no | 1.3886 (1.0431 - 1.8486) | 1.3320 (1.0232 - 1.7338) | 1.7889 (1.3802 - 2.3187) | 1.5238 (1.1406 - 2.0357) |
| surgery: previous year vs no surgery | 1.5413 (0.8828 - 2.6910) | 1.1785 (0.6677 - 2.0801) | 1.3653 (0.8233 - 2.2641) | 1.5987 (0.9156 - 2.7913) |
| surgery: 1 to 5 years ago vs no surgery | 1.1186 (0.7238 - 1.7288) | 1.1649 (0.7766 - 1.7472) | 1.3022 (0.8817 - 1.9233) | 1.2596 (0.8161 - 1.9441) |
| surgery: 5+ years ago vs no surgery | 0.8390 (0.5260 - 1.3385) | 0.8103 (0.5295 - 1.2400) | 1.0271 (0.6833 - 1.5438) | 1.1151 (0.7056 - 1.7624) |
| biological treatment: currently vs no | 1.1301 (0.8252 - 1.5477) | 0.8177 (0.6086 - 1.0986) | 1.0298 (0.7703 - 1.3767) | 1.3602 (0.9859 - 1.8766) |
| active disease vs remission | 2.3833 (1.7813 - 3.1887) | 2.4701 (1.8686 - 3.2652) | 1.1337 (0.8463 - 1.5189) | 2.6078 (1.9397 - 3.5060) |
| UC vs CD or other IBD | 1.4470 (0.6034 - 3.4700) | 1.9861 (1.4800 - 2.6653) | 2.3177 (1.7627 - 3.0474) | 1.4199 (0.2660 - 7.5791) |
| Bulgaria vs Belgium | 0.6857 (0.2567 - 1.8316) | 1.2758 (0.5530 - 2.9431) | 1.7785 (0.6867 - 4.6065) | 2.3700 (0.4868 - 11.5371) |
| Cyprus vs Belgium | 1.9548 (0.5849 - 6.5335) | 1.8903 (0.4584 - 7.7947) | 1.4980 (0.2958 - 7.5867) | 1.1718 (0.1926 - 7.1300) |
| Czech Republic vs Belgium | 0.1445 (0.0434 - 0.4814) | 0.3436 (0.1367 - 0.8633) | 1.2755 (0.4670 - 3.4833) | 1.0136 (0.2119 - 4.8474) |
| Denmark vs Belgium | 0.0550 (0.0281 - 0.1076) | 0.4723 (0.2534 - 0.8806) | 0.8771 (0.4236 - 1.8162) | 1.1579 (0.4453 - 3.0107) |
| Greece vs Belgium | 0.7012 (0.3509 - 1.4010) | 0.5929 (0.2834 - 1.2403) | 0.7932 (0.3255 - 1.9328) | 0.3494 (0.1076 - 1.1348) |
| Spain vs Belgium | 0.2036 (0.0835 - 0.4964) | 0.4759 (0.2400 - 0.9438) | 0.8114 (0.3496 - 1.8830) | 1.2092 (0.3924 - 3.7261) |
| Poland vs Belgium | 0.8769 (0.3819 - 2.0135) | 1.4718 (0.7499 - 2.8886) | 2.0731 (0.9353 - 4.5950) | 1.0132 (0.3371 - 3.0457) |
| Portugal vs Belgium | 0.3674 (0.2018 - 0.6687) | 0.4092 (0.2210 - 0.7574) | 0.7233 (0.3355 - 1.5593) | 1.7496 (0.6576 - 4.6553) |
| Romania vs Belgium | 1.7553 (0.5415 - 5.6902) | 1.5308 (0.6436 - 3.6412) | 5.6996 (2.1225 - 15.3054) | 5.2292 (1.3423 - 20.3720) |
| Hungary vs Belgium | 0.3428 (0.1158 - 1.0149) | 0.5854 (0.2539 - 1.3498) | 1.2874 (0.4390 - 3.7750) | 3.9713 (1.1969 - 13.1764) |
| Italy vs Belgium | 0.6461 (0.2554 - 1.6347) | 0.5297 (0.2395 - 1.1714) | 1.8768 (0.7608 - 4.6295) | 1.3301 (0.3789 - 4.6689) |
| UC#Bulgaria vs CD#Belgium | 0.5192 (0.1239 - 2.1763) | - | - | 0.6222 (0.0652 - 5.9405) |
| UC#Cyprus vs CD#Belgium | 0.6609 (0.0422 - 10.3507) | - | - | 0.0000 (0.0000 - 0.0000) |
| UC#Czech Republic vs CD#Belgium | 0.1588 (0.0121 - 2.0820) | - | - | 0.7559 (0.0497 - 11.4993) |
| UC#Denmark vs CD#Belgium | 0.4286 (0.1402 - 1.3105) | - | - | 0.7357 (0.1309 - 4.1333) |
| UC#Greece vs CD#Belgium | 1.0986 (0.3245 - 3.7189) | - | - | 0.9010 (0.0994 - 8.1653) |
| UC#Spain vs CD#Belgium | 0.6595 (0.1602 - 2.7142) | - | - | 1.0425 (0.1487 - 7.3092) |
| UC#Poland vs CD#Belgium | 0.6872 (0.2053 - 2.3003) | - | - | 0.9599 (0.1546 - 5.9605) |
| UC#Portugal vs CD#Belgium | 1.0452 (0.3924 - 2.7838) | - | - | 0.7171 (0.1213 - 4.2407) |
| UC#Romania vs CD#Belgium | 0.7309 (0.1380 - 3.8701) | - | - | 1.1161 (0.1248 - 9.9777) |
| UC#Hungary vs CD#Belgium | 0.3599 (0.0484 - 2.6744) | - | - | 0.2855 (0.0188 - 4.3411) |
| UC#Italy vs CD#Belgium | 0.6440 (0.1428 - 2.9050) | - | - | 1.0055 (0.1258 - 8.0379) |
| Occupational activity: yes vs no | 0.9968 (0.7324 - 1.3567) | 0.9414 (0.7156 - 1.2386) | 0.8414 (0.6411 - 1.1043) | 0.8900 (0.6583 - 1.2033) |
| /cut1 | -0.7761 (-1.5949 - 0.0428) | -1.5298 (-2.3399 - -0.7196) | -1.1851 (-2.1021 - -0.2681) | 1.1601 (0.0289 - 2.2912) |
| /cut2 | 0.9318 (0.1077 - 1.7560) | 0.9493 (0.1412 - 1.7574) | 0.8058 (-0.1087 - 1.7202) | 3.6915 (2.5246 - 4.8584) |
| /cut3 | 2.0510 (1.2159 - 2.8860) | 2.3886 (1.5623 - 3.2149) | 1.9027 (0.9778 - 2.8275) | 5.0174 (3.7703 - 6.2645) |
| /cut4 | 2.9438 (2.0710 - 3.8165) | 3.4036 (2.5532 - 4.2539) | 2.7781 (1.8401 - 3.7162) | 6.2788 (4.8219 - 7.7357) |
| /cut5 | 3.7062 (2.7708 - 4.6416) | 4.2332 (3.3357 - 5.1308) | 3.5750 (2.6093 - 4.5407) | 6.8837 (5.2622 - 8.5053) |
| /cut6 | 4.4027 (3.3875 - 5.4180) | 4.9515 (3.9621 - 5.9408) | 4.1986 (3.2033 - 5.1939) | 7.4935 (5.5825 - 9.4046) |
| /cut7 | 5.0569 (3.8754 - 6.2385) | 5.4156 (4.3229 - 6.5083) | 5.0255 (3.9167 - 6.1343) | 8.5964 (5.7512 - 11.4417) |
| Pseudo R2 | 0.157 | 0.0565 | 0.0625 | 0.0779 |

Supplementary Figure 1.

Raw frequencies of categories of monthly out-of-pocket expenses on consultations with a specialist among patients with Crohn disease (CD) in remission (A), active CD (B), ulcerative colitis (UC) in remission (C) and active UC (D).

Supplementary Figure 2.

Raw frequencies of categories of monthly out-of-pocket expenses on medications prescribed or recommended by physicians among patients with Crohn disease (CD) in remission (A), active CD (B), ulcerative colitis (UC) in remission (C) and active UC (D).

Supplementary Figure 3.

Raw frequencies of categories of monthly out-of-pocket expenses on dietary supplements, special diet, special equipment, ostomy pouches, transportation to the medical facility among patients with Crohn disease (CD) in remission (A), active CD (B), ulcerative colitis (UC) in remission (C) and active UC (D).

Supplementary Figure 4.

Raw frequencies of categories of monthly out-of-pocket expenses on informational materials about the disease, additional hygiene products and others among patients with Crohn disease (CD) in remission (A), active CD (B), ulcerative colitis (UC) in remission (C) and active UC (D).

Supplementary Table 3.

The raw out-of-pocket patient expenses per patient per month by country, disease type (Crohn disease, CD or ulcerative colitis, UC) and disease activity (raw mean; in 2019 euros).

| Country | IBD | ‘Consultations’ |  | ‘Medications’ |  | ‘Supplements’ |  | ‘Other expenses’ |  |
| --- | --- | --- | --- | --- | --- | --- | --- | --- | --- |
|  |  | Remission | Active disease | Remission | Active disease | Remission | Active disease | Remission | Active disease |
| BE | CD | 31.18 | 65.85 | 29.41 | 76.43 | 40.95 | 54.89 | 20.48 | 16.07 |
|  | UC | 51.96 | 63.75 | 43.60 | 63.75 | 38.42 | 56.00 | 2.78 | 0.00 |
| BG | CD | 29.36 | 53.34 | 59.91 | 76.46 | 41.79 | 86.33 | 20.48 | 35.62 |
|  | UC | 21.65 | 47.29 | 57.06 | 93.67 | 43.71 | 62.27 | 16.04 | 20.75 |
| CY | CD | 52.00 | 87.82 | 63.41 | 54.75 | 49.85 | 78.36 | 9.09 | 19.71 |
|  | UC | 66.50 | 110.89 | 66.55 | 137.15 | 56.06 | 87.20 | 14.54 | 14.62 |
| CZ | CD | 2.78 | 25.67 | 18.69 | 43.83 | 24.94 | 69.42 | 8.28 | 24.18 |
|  | UC | 4.24 | 11.89 | 23.18 | 41.50 | 19.61 | 68.46 | 12.74 | 19.55 |
| DK | CD | 4.51 | 13.41 | 23.08 | 45.99 | 25.01 | 47.03 | 8.79 | 20.33 |
|  | UC | 0.00 | 6.25 | 50.41 | 64.83 | 26.32 | 51.58 | 7.69 | 18.71 |
| ES | CD | 17.03 | 31.38 | 26.21 | 49.56 | 27.82 | 58.22 | 8.33 | 24.35 |
|  | UC | 14.25 | 12.50 | 36.80 | 47.06 | 29.47 | 33.86 | 11.61 | 23.44 |
| GR | CD | 38.41 | 52.74 | 35.60 | 44.52 | 31.08 | 49.04 | 4.21 | 9.40 |
|  | UC | 47.78 | 77.42 | 50.30 | 66.10 | 38.59 | 60.12 | 17.67 | 18.69 |
| HU | CD | 17.19 | 34.13 | 27.53 | 54.41 | 36.05 | 66.44 | 16.36 | 31.39 |
|  | UC | 21.55 | 13.85 | 30.32 | 29.08 | 42.71 | 20.75 | 14.60 | 20.33 |
| IT | CD | 23.51 | 67.10 | 28.40 | 67.14 | 40.82 | 81.60 | 11.10 | 28.75 |
|  | UC | 32.70 | 49.82 | 31.77 | 86.29 | 40.81 | 92.11 | 9.84 | 36.27 |
| PL | CD | 35.86 | 86.29 | 65.49 | 102.35 | 48.05 | 83.12 | 9.80 | 18.81 |
|  | UC | 43.26 | 72.19 | 63.16 | 87.86 | 56.19 | 79.84 | 10.37 | 18.04 |
| PT | CD | 19.42 | 38.35 | 22.65 | 41.38 | 28.83 | 60.73 | 11.80 | 27.80 |
|  | UC | 29.35 | 98.80 | 31.12 | 58.60 | 20.20 | 49.11 | 11.44 | 33.54 |
| RO | CD | 62.71 | 108.29 | 53.65 | 106.30 | 115.84 | 135.47 | 48.36 | 47.32 |
|  | UC | 55.54 | 104.74 | 61.58 | 107.62 | 74.74 | 113.68 | 32.00 | 66.78 |
